# A Method for Multiplex Gene Synthesis Employing Error Correction Based on Expression

**DOI:** 10.1371/journal.pone.0119927

**Published:** 2015-03-19

**Authors:** Timothy H.-C. Hsiau, David Sukovich, Phillip Elms, Robin N. Prince, Tobias Stritmatter, Paul Ruan, Bo Curry, Paige Anderson, Jeff Sampson, J. Christopher Anderson

**Affiliations:** 1 Department of Bioengineering, University of California, Berkeley, CA, United States of America; 2 Department of Electrical Engineering and Computer Science, University of California, Berkeley, CA, United States of America; 3 Agilent Technologies, Santa Clara, CA, United States of America; 4 Synthetic Biology Institute, University of California, Berkeley, CA, United States of America; Deutsches Krebsforschungszentrum, GERMANY

## Abstract

Our ability to engineer organisms with new biosynthetic pathways and genetic circuits is limited by the availability of protein characterization data and the cost of synthetic DNA. With new tools for reading and writing DNA, there are opportunities for scalable assays that more efficiently and cost effectively mine for biochemical protein characteristics. To that end, we have developed the Multiplex Library Synthesis and Expression Correction (MuLSEC) method for rapid assembly, error correction, and expression characterization of many genes as a pooled library. This methodology enables gene synthesis from microarray-synthesized oligonucleotide pools with a one-pot technique, eliminating the need for robotic liquid handling. Post assembly, the gene library is subjected to an ampicillin based quality control selection, which serves as both an error correction step and a selection for proteins that are properly expressed and folded in *E*. *coli*. Next generation sequencing of post selection DNA enables quantitative analysis of gene expression characteristics. We demonstrate the feasibility of this approach by building and testing over 90 genes for empirical evidence of soluble expression. This technique reduces the problem of part characterization to multiplex oligonucleotide synthesis and deep sequencing, two technologies under extensive development with projected cost reduction.

## Introduction

Despite impressive cost improvements in commercial gene synthesis over the last decade, cost of synthetic DNA remains a limiting factor for experiments in the laboratory. A common desire when engineering organisms is to mine homologous genes for the best functional performer in a heterologous host organism. Gene expression from synthetic DNA allows for codon optimization for expression in the new host, and circumvents collection of genomic DNA for templates. However, the cost to synthesize 100 genes 1000 bp in length sums to $20,000 at $0.20/bp, making this a relatively expensive experiment. Additionally, the space to mine for a given function is expanding as more sequenced genomes become available.

Commercial methods for gene synthesis rely on assembly of short singled-stranded DNA oligonucleotides into a full-length double-stranded synthon. Microarray oligonucleotide library synthesis (OLS) technology is a cheap source of DNA, providing thousands of oligonucleotides per array. Gene synthesis methods utilizing OLS DNA either amplify specific oligonucleotides required for assembly of one gene from the library background [1], or utilize methodology to isolate specific DNA sequences [2,3]. However, when assembling hundreds of genes, pooling specific oligonucleotides per gene requires liquid handling robotics to be practical, making it out of reach for the average laboratory. Oligonucleotide synthesis is also imperfect (2–2.7 errors/kb [1,4]), resulting in errors in assembled genes. Enzymatic error correction methods are utilized to enrich for perfect gene sequences by cleaving mismatches in the annealed oligonucleotides [5]. However, DNA sequencing is still required to select perfect clones, making quality control a laborious part of the process.

To make synthesis of open reading frame variants more practical and accessible by reducing both the cost and labor required, we propose linking a selection for functional characterization to a simplified one-pot gene synthesis protocol. As only properly assembled genes are functional, this not only serves as an error correction method, but also identifies and enriches for the best performers out of the gene library. We have developed a protocol capable of assembling 100 genes ~800 bp in length in one tube, trading an increase in error rate for a greatly simplified liquid handling. We linked our library of synthesized genes to the general need for proper protein folding and expression of the heterologous genes in *E*. *coli* as our functional characterization selection parameter, resulting in 60% perfect clones. The best performing genes were identified from the library via deep sequencing, removing the need for quality control on correctly synthesized library members that were improperly folded or poorly expressed. All together, this reduces experimental cost to microarray OLS DNA and next generation sequencing reagents, totaling around $2000. We refer to this technique as Multiplex Library Synthesis and Expression Correction (MuLSEC), a high-throughput one-pot gene synthesis protocol followed by *E*. *coli* solubility based selection error correction.

## Results

### Multiplex synthesis of individual genes

**Fig 1:**
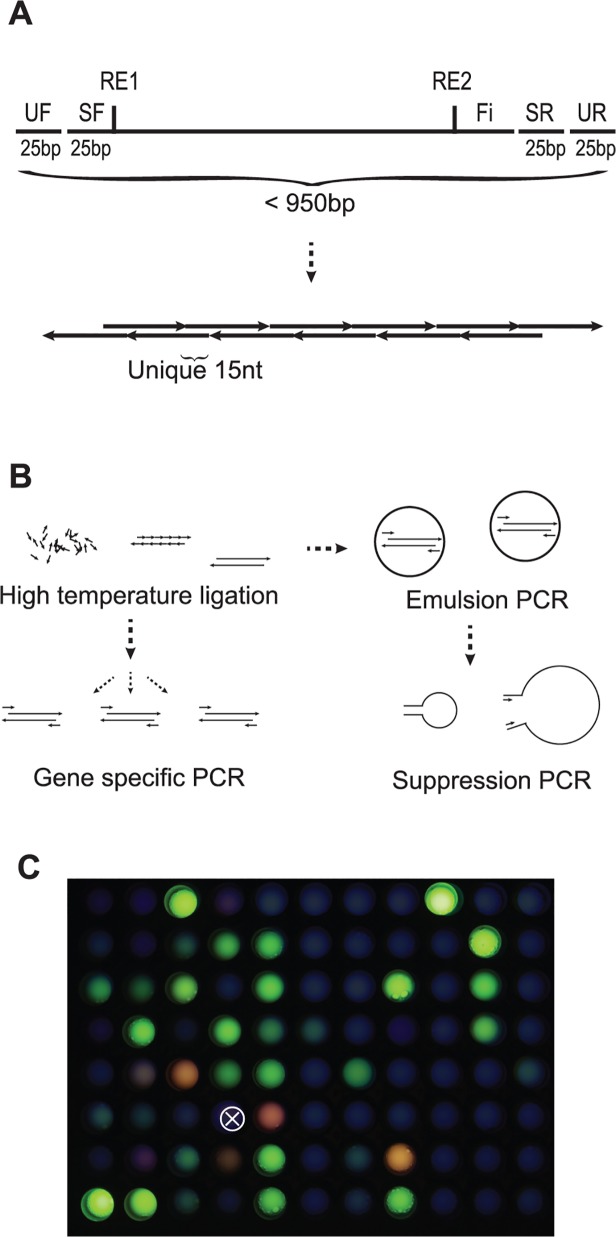
Multiplex Library Synthesis of Genes. (A) Individual genes (top) are assembled from a set of oligonucleotides (arrows) with 15 bp of unique overlapping sequence. Synthons include universal forward (UF) and reverse (UR) primer binding regions common for all genes in the library, and specific forward (SF) and reverse (SR) sites for amplification of individual genes. Synthons include restriction endonuclease sites (RE1 = EcoRI, RE2 = BamHI) for downstream cloning purposes, a random filler sequence (Fi) for length standardization, and are < 950 bp long. (B) Gene synthesis from a pooled library of oligonucleotide starts with high temperature annealing and ligation, then individual genes can either be amplified with SF/SR primers (left) or library of genes is synthesized and amplified (right) with emulsion PCR using UF/UR primers with annealing 5’ and 3’ overhangs to enable suppression PCR, which preferentially amplifies longer synthons. (C) Excitation of 87 GFP variants successfully synthesized, amplified individually, then expressed in *E*. *coli* show functional fluorescent protein at various emission wavelengths. Images are combined from ultraviolet (top layer, 50% transparency) and blue light illumination (470nm) with no emission filtering. White X represents the negative control well with media only.

To simplify the liquid handling required for assembly of several individual genes from OLS libraries, we aimed to assemble a pooled library of genes all in one multiplexed reaction. We chose 89 green fluorescent protein (GFP) family members selected from UniProt [6] and 16 other random sequences for a total of 105 sequences as our test library. Genes were codon optimized for *E*. *coli*. with a choice weight proportional to *E*. *coli* codon frequencies. Synthon length was standardized at 711bp by addition of random padding sequences at the 3’ end (GC ~50%) and restriction enzyme sites for EcoRI and BamHI were added flanking each gene to enable cloning into an expression vector. Gene specific and universal primer sequences were added on the ends of each synthon [1,7,8] to allow for PCR amplification of the entire library or each gene individually. The total DNA assembly length was kept under 950 bp (Fig. 1A).

Oligonucleotides to assemble the library of genes were designed to have fifteen nucleotides of unique sequence at both termini to minimize cross talk between each gene assembly reaction and overlapping regions were selected such that the melting temperature was 65°C. The designed oligonucleotides ranged between 33 and 137 nt, with a median length of 62 nt. The OLS chip for this design contained 2,726 oligonucleotides, resulting in 169 kb of sequence. Oligonucleotides were synthesized by Agilent and received as a multiplex pool. Oligonucleotides were phosphorylated, then assembled into full-length genes with a thermostable DNA ligase at 65°C (Fig. 1B). Individual genes were PCR amplified from the multiplexed gene synthesis reaction with specific forward and reverse primer pairs and 99 of 105 sequences had a PCR band. PCR products were then subcloned into an expression vector. The robustness of the protocol was demonstrated when correctly-sized PCR products were observed even when 1 picomole of a 25 nt random oligonucleotide (N_25_) was doped into the ligation reaction (20 pM of microarray oligos). The 16 random sequences were ignored for further analysis and gene synthesis products for each gene were screened by picking fluorescent colonies or with Sanger DNA sequencing for genes with no detectable fluorescence. Correct products were found for 85 of the 89 GFP genes. The remaining four genes were fluorescent but Sanger sequencing revealed they had point mutations. From randomly picked non-fluorescent colonies, the frequency of correct colonies was determined to be 15% (6/40), with the majority of errors being point deletions, as is expected from the oligonucleotide synthesis methodology [9]. Several library members exhibit fluorescence with different emission wavelengths when excited at 470 or 365 nm (Fig. 1C).

We next tested library designs to either increase the number of genes synthesized per multiplex reaction (1000 genes 800 bp long), or increase the synthon length (200 genes 1500 bp long). However, we were unable to successfully assemble either design, as only partial fragments were recovered for the longer and more complex gene library and no perfect sequences were recovered for a majority of genes in the thousand gene library. We did not attempt a design of 100 genes of 1500 bp in length but instead continued with the proven design of a ~100 synthons of ~800 bp in length.

### Multiplex synthesis of a gene library

We next sought to enable a one-pot process by eliminating the need for post assembly gene-specific amplification. Our initial attempts to directly amplify the pool of assembled genes using universal forward and reverse primers from the complex ligation assembly reaction using conventional PCR yielded only short (100–200 bp) products. We reasoned that the shorter products were favored by the PCR reaction and sought to counter or invert the length bias of PCR. To counteract this, we applied amplification by emulsion [10] and suppression PCR [11]. Water-in-oil emulsion PCR with universal forward and reverse primers was carried out on the ligation reaction first and generated a faint band of the correct size when analyzed on an agarose gel. However, use of emulsion PCR alone was not robust, as occasionally no DNA band was observed on a gel, likely due to low reaction yield. Therefore, emulsion PCR was used to add flanking inverted repeat tails to enable suppression PCR. A single primer, which binds to the inverted repeats is used in suppression PCR for amplification. The inverted repeats anneal to each other and compete with primer binding (Fig. 1B). Shorter amplicons exhibit the suppression effect more than longer amplicons, thus suppression PCR is biased towards longer products [11], resulting in correctly sized DNA products when analyzed via gel electrophoresis.

After amplification of all genes as a pooled library, bands of the proper size were excised from a gel and cloned via restriction enzyme digestion into an expression vector. Colonies were randomly picked and Sanger sequenced. The number of correct, full-length genes was 21% (3/14), while 43% (6/14) of clones were fusions of two genes and the remainder of the errors were deletions or truncated genes. We concluded that our one-pot multiplex gene synthesis method was sufficient to quickly create a library of genes.

### A high-throughput expression correction method based on Tat quality control

**Fig 2:**
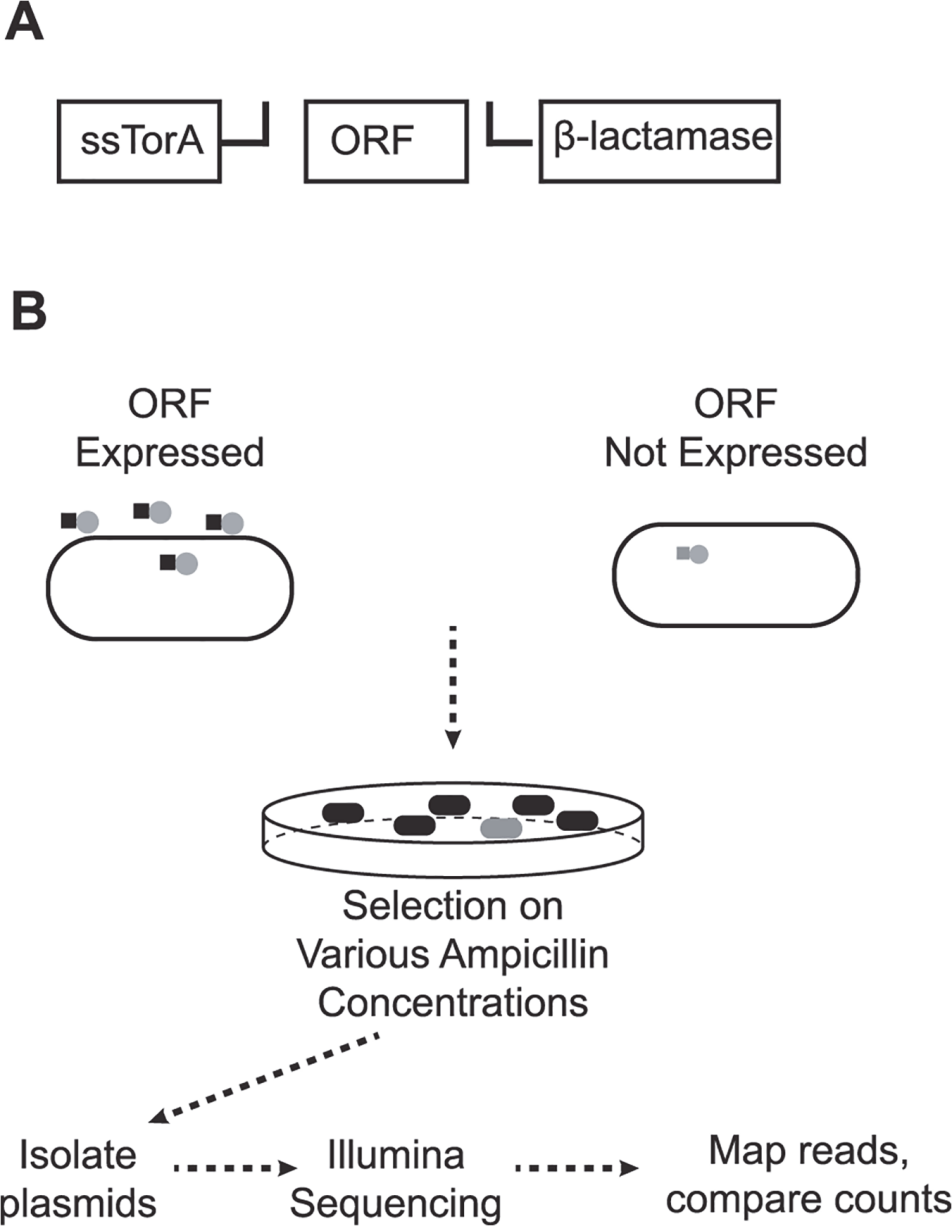
Expression correction for properly folded soluble proteins. (A) The synthesized gene library open reading frames (ORFs) are cloned via RE1/RE2 into a vector containing an N-terminal Tat pathway secretion signal (ssTorA) and a C-terminal TEM-1 β-lactamase. (B) Synthesized genes that are expressed, properly folded, and soluble result in the export of mature β-lactamase fusions to the *E*. *coli* periplasm, which confers ampicillin resistance. Selection against insoluble protein serves to both remove correct gene sequences which are poorly expressed in *E*. *coli* and remove incorrect gene synthesis products as the resulting protein is often insoluble. Expression profiling is characterized with Illumina MiSeq next generation sequencing.

We sought to develop a multiplex expression assay in order to avoid individual cloning and sequence verification to select for correct genes. Recently, a selection assay based on the twin-arginine export quality control mechanism was developed in *E*. *coli* [12]. In this system, the gene of interest is fused at the 5’ end with a Tat export signal derived from trimethylamine N-oxide reductase (ssTorA) and fused at the 3’ end with the mature TEM-1 β-lactamase (Fig. 2A). Only correctly folded proteins translocate to the periplasm and confer resistance to ampicillin [13]. By cloning our synthesized multiplex gene library into an expression vector with N-terminal ssTorA and C-terminal β-lactamase and performing selection on ampicillin, we aimed to not only remove genes that were improperly folded, but also those that were poorly expressed or toxic to *E*. *coli*. Genes with frameshift errors and incomplete fragments were selected against as well, as they are not likely to be folded properly or generate functional C-terminal β-lactamase. Expression correction serves as a quality selection method to reduce gene synthesis associated errors and enrich for genes with good expression characteristics in *E*. *coli*. When coupled with next generation sequencing, the best performing genes are easily identified for downstream experiments (Fig. 2B).

**Fig 3:**
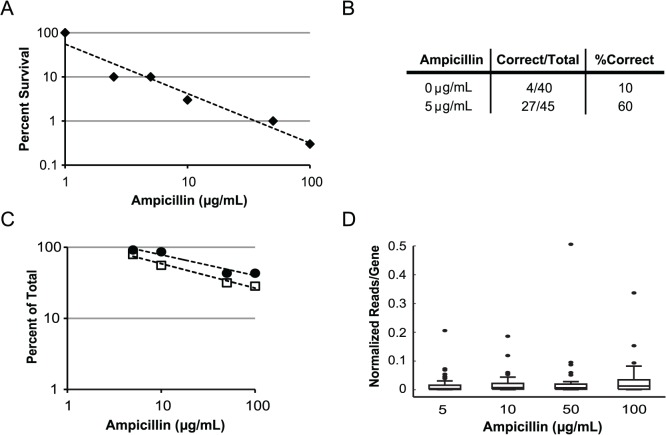
Multiplex Library Synthesis and Expression Correction (MuLSEC) Characterization for a Library of Enzymes. (A) Survival of clones after multiplex library gene synthesis is presented on the y-axis versus the solid media ampicillin concentration on the x-axis. Fewer clones survive on increasing ampicillin concentrations. (log-log plot). (B) Table of Sanger sequencing results on individual clones. Selection on ampicillin at 5 μg/mL led to a 6x increase in the number of perfect clones. (C) Percent of the 95 genes synthesized detected by next generation sequencing after selection on varying concentrations of ampicillin. Data is presented with a cutoff such that the median number of reads per gene must be ≥ 0.05% of total reads per ampicillin concentration condition (open squares) or with no cutoff (closed circles). Fewer genes are detected with increasing ampicillin concentration (log-log plot). (D) Box-and-whisker plot of pool normalized representation of each gene (reads ≥ 0.05% of group total) for increasing ampicillin concentrations. Black dots represent outliers in the dataset.

To test expression correction, we compiled a second gene library consisting of a variety of enzymes to further diversify protein characteristics compared to the pool of GFP homologs. We chose 69 genes less than 711 bp in length from the BRENDA [14] database to represent a wide range of enzyme function, six of which were native *E*. *coli* genes to serve as positive controls. Additionally, we included 7 negative controls previously shown to be poorly expressed in *E*. *coli* [13] and 19 GFP variants for a total of 95 genes. Genes were synthesized from OLS oligonucleotides with our multiplex one-pot protocol then cloned into a vector such that ssTorA was fused to the N-terminus and β-lactamase to the C-terminus (pSALect-EB). After insertion of the gene library into *E*. *coli*, culture was selected on varying concentrations of ampicillin (Fig. 3A). At 1 μg/mL of ampicillin, no drop in colony titer was observed compared with no ampicillin. However, only 10% of these colonies survived at 2.5 and 5 μg/mL. Sanger DNA sequencing of individual clones selected at random resulted in 60% (27/45) correct full-length sequences at 5 μg/mL ampicillin compared to only 10% (4/40) at 0 μg/mL (Fig. 3B).

To characterize the resulting gene library after selection on ampicillin, next generation sequencing was used to quantify representation of each gene after selection at 5, 10, 50 and 100 μg/mL. Plasmid DNA was randomly fragmented, thus the full gene insert is not sequenced in any single read. Resulting reads were mapped to reference genes based on short matching DNA sequences, therefore incorrect gene fragments generated during DNA synthesis are counted as well. However, representation of full-length genes versus gene fragments can be distinguished by quantifying the base-by-base coverage of each gene. Therefore, the median of the base-by-base coverage was chosen as a length-normalized count of representation. A total of 7.9 million reads were generated of which 18.7% mapped to the reference genes. Unique DNA barcodes were assigned to each ampicillin selection condition, allowing quantification of each gene per experimental condition. The sum of base-by-base median of all genes in each pool varied, therefore reads were normalized by the sum of each pool to allow for quantitative comparison between conditions.

At the lowest ampicillin concentration (5 μg/mL), reads were mapped to 87 of the 95 genes (92%). If a representation cutoff is applied such that each median of base-by-base coverage must be ≥ 0.05% of each ampicillin pool, this drops to 75 mapped genes at 79%. As the ampicillin concentration is increased, genes drop out of the pool, with only 27% of genes represented ≥ 0.05% at 100 μg/mL (Fig. 3C). All of the negative solubility controls exhibited a pattern of low representation in the higher ampicillin concentrations, rapidly falling off after 5 μg/mL. Of the 6 *E*. *coli* positive controls, we found that two survived (dgoA and supH) at high ampicillin concentrations up to 100 μg/mL, while the others died at 50 μg/mL or more ampicillin (gmk, lacA, dcd, and pabC). Of the 19 GFP homologs included, the well-folding monomeric mKG [15] was the best performer in the 100 μg/mL pool representing 3.6% of reads. These results suggest that Tat quality control is convenient with no observed false positives and the window separating positives from negatives lies within the range of 5–50 μg/mL of ampicillin.

NGS-predicted expression was correlated with text-mined expression predictions. Text-mined evidence for expression was inferred from the "cloned" commentary section of the BRENDA database[14] counting entries with the organism name "escherichia", and without the terms "inclusion bodies" and "folding" as evidence of expression. Of the 69 BRENDA-derived test genes, 58 (84%) were predicted to be expressed in *E*. *coli* by text mining, while NGS gave 52 (75%) genes in the 5 μg/mL ampicillin pool with a ≥ 0.05% representation cutoff. There are 43 (62%) genes in the intersection of the two sets, suggesting that the text mining approach has 83% true positive and 17% false negative rates.

The majority of genes fell around the median for each normalized pool, representing 0.37% at 5 μg/mL and 1.3% at 100 μg/mL (Fig. 3D). However, a few genes in each pool were outliers, comprising up to one half of the DNA pool. The largest outlier at all ampicillin levels was BLVRB, a flavin reductase from *Bos taurus*. It comprised 21% of the reads at 5 μg/mL and 34% at 100 μg/mL, providing a 1.6 fold enrichment between the two conditions. This could be due to increased growth rate with BLVRB expression or an advantage in gene assembly efficiency during multiplex synthesis. BLVRB was also most represented gene during Sanger sequencing. The ratio between BLVRB and the second ranked HAMI, a nucleoside-triphosphate diphosphatase from *Corynebacterium efficiens*, is comparable between Sanger (2.67) and next generation sequencing (2.82) [8:3 clones with Sanger sequencing versus 3197:1131 with NGS median read coverage] at 5 μg/mL of ampicillin. The most enriched gene at 36.6 fold was *sseA*, a thiosulfate sulfurtransferase from *Azotobacter vinelandii*, increasing from 0.2% to 7.3% of the pool between 5 and 100 μg/mL of ampicillin.

### Phenotypic and biochemical characterization of a subset of synthesized individual genes

**Fig 4:**
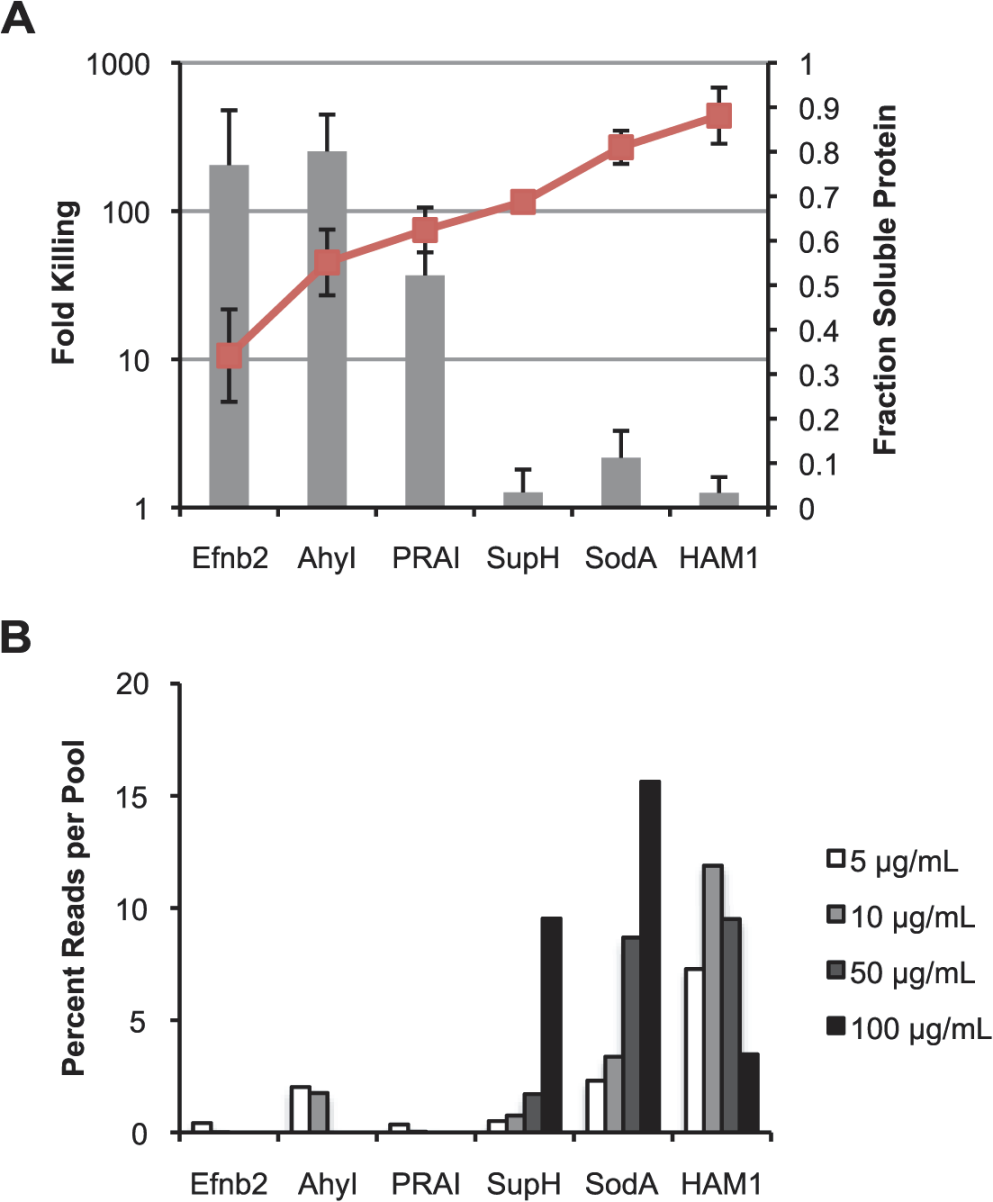
Individual Gene Performance in Expression Correction Assay on Ampicillin. (A) Fold killing on 100 μg/mL of ampicillin (left axis, gray bars) and fraction of soluble protein (right axis, red line) for each individual gene shows an increase in cell survival with increasing soluble protein fraction. Fold killing is calculated based on viable colony counts on solid media with no ampicillin divided by counts at 100 μg/mL ampicillin. Soluble protein fraction was quantified from western blotting of soluble and insoluble protein fractions expressed as C-terminal FLAG tag fusions. (n = 3 for both experiments). Error bars represent standard deviation. (B) Percent representation of each gene per ampicillin selection pool shows enrichment of genes which survive at 100 μg/mL of ampicillin and have higher soluble protein fractions in (A). Percent is calculated as the median number of reads per base pair of each gene compared to the sum of medians for each gene per ampicillin pool.

Five BRENDA enzymes from various prokaryotes and the insoluble negative control mouse protein, Efnb2, were chosen for further individual phenotypic characterization (Table 1). In order to assess soluble and insoluble protein, each was cloned with a C-terminal FLAG tag to enable Western blotting of insoluble and soluble protein fractions. This demonstrated gene-to-gene variation in both total protein expression and the distribution between the soluble and insoluble fractions (S1 Fig.). The expression level of each in the soluble fraction was measured by quantitative image analysis of western band intensities, normalizing the soluble fraction by the total protein expression, calculated as the sum of the insoluble and soluble fractions. Additionally, fold killing was quantified by counting viable colonies with the same six genes expressed as ssTorA/ β-lactamase fusions on ampicillin at 0 versus 100 μg/mL (S2 Fig.). When ordered from lowest to highest soluble protein fraction, fold killing also decreased sharply at soluble protein fractions > 0.62 (Fig. 4A). The negative control, Efnb2, and enzyme AhyI had the highest fold killing on ampicillin at > 200x, and also had the lowest fraction soluble protein at 0.34 and 0.55, respectively. The enzymes SupH, SodA and HAMI survived on 100 μg/mL of ampicillin and also had the highest soluble protein fractions at 0.69 or greater. PRAI existed as an intermediate phenotype for both soluble protein fraction and fold killing with 37x cell death on ampicillin and a soluble protein fraction of 0.62. Therefore proteins with higher soluble protein fraction confer survival on ampicillin, which is consistent with previous work[12].

**Table 1:** Enzyme Library Genes Assayed for Individual Performance Analysis.

Name	Organism	EC#	Function
Efnb2	*Mus musculus*	—-	cell surface transmembrane ligand
AhyI	*Aeromonas hydrophila*	2.1.1.184	acyl-homoserine-lactone synthase
PRAI	*Thermotoga maritima*	5.3.1.24	phosphoribosylanthranilate isomerase
SupH	*Escherichia coli*	3.1.3.23	sugar phosphatase
SodA	*Geobacillus stearothermophilus*	1.15.1.1	superoxide dismutase
HAM1	*Cornynebacterium efficiens*	3.6.1.15	nucleoside-triphosphate phosphatase

Five genes from the set of 69 BRENDA enzymes and one control (Efnb2) selected for performance in the expression correction ampicillin-based selection. Efnb2 was previously shown to be insoluble and die as a ssTorA/ β-lactamase fusion on ampicillin[11]. EC refers to enzyme classification number.

Normalized median read count for each of the six genes from the next generation sequencing assay were compared at 5, 10, 50, and 100 μg/mL of ampicillin (Fig. 4B). Proteins Efnb2, AhyI and PRAI with intermediate to high degrees of killing on ampicillin were not present in the DNA pool of next generation sequencing reads at 50 and 100 μg/mL. However, enzymes SupH, SodA, and HAMI were well represented at higher concentrations. SodA and SupH both showed enrichment in the number of reads as ampicillin concentration is increased from 0 to 100 μg/mL at 6.6 and 18.4, respectively. However, HAMI reads remained relatively constant as ampicillin concentration was increased, even dropping below the level at 5 μg/mL at 50 and 100 μg/mL. Therefore, despite constituting 3.5% of the DNA reads at 100 μg/mL, HAMI enrichment from 5 to 100 μg/mL was less than one at 0.47. We hypothesize the lack of enrichment was due to retarded growth rates of HAMI expressing *E*. *coli* compared to SodA and SupH in the presence of ampicillin. Despite this outlier, the data demonstrates that next generation sequencing enrichment compares well to phenotypic analysis of survival on ampicillin and soluble protein fraction.

## Methods

### Design of synthesized sequences

All GFP family members from Uniprot were assembled into a phylogenetic tree and 89 variants were selected to represent the diversity of sequences available. The additional 15 sequences were generated by ancestral sequence reconstruction but were not cloned or studied for fluorescence. The BRENDA enzyme set was randomly selected from genes that were less than 1 kb in length. Protein sequences were converted to nucleotides using a weighted random codon algorithm designed in-house that excluded designs with internal EcoRI and BamHI sites. Oligonucleotides to assemble the genes were designed to be ≤ 175 nt in length with overlapping regions selected such that the melting temperature was 65°C. Additionally, no more than 15 nucleotides could match between oligonucleotides at either terminus. All gene and oligo sequences can be found in the Supplementary Materials.

### Gene synthesis by high-temperature ligation

Oligonucleotides were synthesized by Agilent and were received resuspended in 100 μL of TE buffer. Oligonucleotides were phosphorylated using 3 μL T4 Ligase Buffer (NEB), 24 μL OLS oligonucleotides, and 3 μL T4 PNK(NEB) at 37°C for 1 hour. The reaction was heat inactivated at 65°C for 30 min and held at 16°C. Whole pool ligation was performed with 12 μL phosphorylated oligonucleotides, 4 μL 50% 3350 PEG (Carbowax P146–3), 2 μL 9°N buffer, 2 μL (80 U) of 9°N ligase (NEB). Reactions were performed in a MJ Research PTC-200 thermocycler using the following program: 95°C for 2 minutes, 65°C for 24 hours, and 4°C hold. The ligation product was used as template for gene-specific PCRs or emulsion PCR. Gene-specific PCR was performed using 0.25 μL of the ligation product as template with gene-specific primers. Testing several commercially available thermostable ligases revealed no differences in the gene-specific PCR for a subset of 20 genes.

### Emulsion PCR for post-ligation amplification

Emulsion oil mix was prepared with 450 μL Span 80 (Fluka 85548), 40 μL Tween 80 (Sigma P4780), 5 μL Triton X-100 (Promega H5142), and 9505 μL mineral oil (Sigma M5904) as described in [9] and was thoroughly vortexed to mix. Separately, a PCR reaction mix was prepared on ice using Q5 polymerase (NEB). PCR reactions were performed using 10 μL of ligation product as template supplemented with 0.5 μL (1 U) of Q5 polymerase, 20 μL Q5 reaction buffer, 1 mM dNTP, and water for a total reaction volume of 100 μL. For emulsification, PCR reactions were mixed with oil at a 1:10 (PCR:oil) volumetric ratio. The PCR mix was pipetted into a cryovial tube containing emulsion oil and vortexed at maximum power using a VWR benchtop vortexer for 1 minute until a milky white emulsion formed. The emulsion was distributed as 100 μL aliquots and PCR was performed in a MJ Research PTC-200 thermocycler. Aliquots were transferred to microcentrifuge tubes and centrifuged for 20 minutes to separate the oil and aqueous phases, then excess oil was removed from the top. To break the emulsion, 300 μL of 2-butanol (Sigma-Aldrich 19440) was added and tubes were vortexed to mix. To clean up the reaction, 1 mL of ADB (Zymo Research) was added, tubes were vortexed, and PCR clean-up columns (Zymo Research) were used to purify the amplicons. Resulting DNA was visualized using agarose gel electrophoresis and either cloned or further amplified with suppression PCR.

### Suppression PCR to generate clonable amplicons

Purified emulsion PCR products were used as a template for suppression PCR. Both emulsion primers were designed with a suppression tail of (CATCAGGTTTCATCCTGCCGGCATGAGCGGCTAACGG) so that amplicon ends form an inverted repeat. For suppression PCR, the distal-binding primer (CATCAGGTTTCATCCTGCCGG) was used (30 cycles, Tm of 55°C). PCR products were visualized on a gel and the band of the appropriate length was excised and cloned into a vector using EcoRI and BamHI restriction enzymes.

### Solubility assay using a beta-lactamase folding reporter

The pSALect vector[12] was modified to create pSALect-EB by placing EcoRI and BamHI restriction sites between the tat signal and TEM-1 beta-lactamase. For library creation, digested pSALect-EB vector and amplicons were ligated and purified with a PCR clean-up column (Zymo Research). Purified plasmids were then introduced into MC1061 derivative strains by electroporation[16]. Cells were rescued for 2 hours at 37°C and grown overnight in 200 mL 2YT liquid media supplemented with 25 μg/mL chloramphenicol. Post rescue, cells were also serially diluted and plated onto LB chloramphenicol plates for library titering. A dilution equivalent of 1 μL overnight culture was then spread on LB plates supplemented with chloramphenicol (25 μg/mL) and ampicillin at different concentrations ranging from 1 to 100 μg/mL. Plates were incubated for 16 hours at 30°C. Plasmid DNA was isolated from libraries of cells harvested from plates or colonies of single cultures grown overnight to saturation.

### Excitation of GFP variants

Eighty-seven of the correctly synthesized GFP variants were transformed into *E*. *coli* and grown in liquid culture to saturation. Culture (1 mL) was concentrated by centrifugation, and resuspended in PBS, then 200 μL was transferred to a clear bottom 96-well plate with opaque siding. Images were captured with ultraviolet (365 nm) and blue light (470 nm) excitation individually, and no emission filter. Images were merged in Photoshop (Adobe) with a top layer, 50% transparency applied to the ultraviolet image.

### Next generation sequencing of libraries

Plasmid DNA concentration was measured with a Nanodrop (Thermo Scientific). Libraries were prepared using the TruSeq sample preparation kit (Illumina) following the manufacturer’s protocols. Pools were prepared separately, barcoded, and quantified with a Library Quant Kit (Kapa Biosystems). Pools were then combined, and sequenced on a MiSeq (Illumina) using a 300 cycle v2 kit (Illumina). Resulting reads were quality trimmed and mapped to the reference sequences using BWA (0.6.1-r104). Samtools mpileup was used to extract per-base coverage and then a custom python script and Microsoft Excel were used to normalize the data. As some inserts were partial gene fragments and also contributed to the per-base read coverage score, we took the median as the read coverage score for the entire gene. Manual inspection of the read coverage for several genes showed that the median was an acceptable measurement of whole gene read coverage. The read coverage per gene was then pool-normalized by dividing by the sum of read coverages for all genes in each pool. Median counts are provided in an Excel file in the Supplementary Materials.

### Western blotting of soluble and insoluble protein

Plasmids from the 5 μg/mL ampicillin condition were digested with EcoRI and BamHI and the 700 bp band corresponding to the library of gene inserts was gel purified and cloned into an arabinose-inducible expression vector with a C-terminal 3x FLAG tag. Plasmid DNA was isolated from 72 colonies, and Sanger sequencing revealed 26 unique gene inserts were recovered. Six of those genes were selected to be a representative range of ampicillin resistance and cells were transformed with plasmid DNA and the resulting strains were grown overnight, reinoculated, induced with arabinose (0.2% w/v), and harvested after 4 hours. Cells were pelleted by centrifugation for 5 minutes at 2500 rcf, and the cell pellet was resuspended with BugBuster MasterMix (Novagen) at a ratio of 1 mL BugBuster per 0.1 g cell pellet. Cells were lysed for 20 minutes at 25°C on a rocking platform and soluble protein was recovered by taking the supernatant after centrifugation at 12,000 rcf for 15 minutes. The insoluble fraction was resuspended in an equal amount of BugBuster. Subsequent Western blotting was performed with Monoclonal Anti-FLAG M2-HRP antibody (Sigma A8592) and ECL Western Blotting Substrate (Pierce 32106). Band intensity was quantified with ImageJ from digital images, and soluble protein fraction was calculated as the soluble band intensity divided by the sum of the insoluble and soluble band intensities.

## Discussion

We developed a new methodology called MuLSEC, for rapidly building genes and characterizing their soluble expression. We simplified the process of fabricating genes from complex microarray oligonucleotide pools to a method which requires only a one-pot reaction and is able to create genes with length 811 bp at the hundred-scale with a good rate of success (>95% of genes). These results enable researchers to perform only 5-steps, including bulk phosphorylation, ligation, emulsion PCR, suppression PCR, and restriction enzyme based cloning, to make a pool of ~100 genes as opposed to hundreds of steps required by previous protocols. With the use of the ssTorA/β-lactamase fusion system and selection on ampicillin, we have shown it is possible to perform non-enzymatic gene synthesis error reduction in a pooled format while simultaneously selecting for genes that are properly expressed in *E*. *coli*.

When combined with expression correction, multiplex library synthesis is limited to synthesis of open reading frames. Therefore, it is not a replacement for standard commercial DNA synthesis, but rather an efficient and cost effective technique when experimentally mining a library of proteins. Here we demonstrate MuLSEC for relatively short genes < 811bp in length. The median gene length of prokaryotes is 924 bp and 1346 bp for eukaryotes [17], making more than half of the genes inaccessible. However, the technique may be adopted for longer synthons by either reducing the number of genes synthesized per library or breaking up multi-domain proteins into their individually folding subunits combined with an additional round of assembly into a full-length gene.

Our motivation to incorporate expression testing with multiplex library synthesis was the observation that improper expression, folding, or toxicity of heterologous genes often complicate engineering efforts in synthetic biology [18]. Therefore, we sought a method to rapidly assess the feasibility of using particular genes based on their expression characteristics. Experienced researchers empirically learn patterns in the relationship between expression and classes of enzymes and avoid certain designs. For example, it is widely observed that P450 enzymes will not express in *E*. *coli* and this is taken into account when choosing the chassis for a pathway that involves such an enzyme. However, the documented data on heterologous gene expression is sparse; there are 54,247,468 protein entries in UniProt, but only 30,563 expression observations in BRENDA, the largest database containing such data. Additionally, amongst a set of orthologs, some variants will express while others will not. For example, of six leucyl-tRNA synthetases cloned from archeal genomes, only three express in *E*. *coli*[19]. When researchers are only aware of one instance of a class that fails to express, they may incorrectly assume that the design cannot be instantiated. The lack of extensive characterization data leads researchers to expend resources on designs that cannot work, and miss opportunities due to false expectations that a design will not work. With more cost effective and scalable means of empirically obtaining this information, design decisions can be made more precisely and reliably in the planning process.

Our multiplex expression test uses the Tat export pathway and a β-lactamase folding reporter in conjunction with next-generation sequencing to quickly assay expression of hundreds of genes using simple plate-based selections. Using the Tat export pathway allows our expression system to avoid false positives arising from translation due to spurious ribosomal binding sites internal to the assayed ORF; however, it also confers some disadvantages on our system. Fusing domains is known to make some proteins insoluble. The Tat pathway has a limit on the size of proteins it can export, and while proteins of up to 120 kDa have been shown to be exported[13], the full scope of factors that influence what can and cannot be exported are unknown. Multimeric complexes such as wild-type dsRed have also been shown to not efficiently transit the Tat pathway[12]. However, complementary approaches could be implemented in parallel to reduce the error rate associated with limitations of the Tat system, such as GFP fusions where fluorescence correlates with the solubility of the fusion partner [20], loop insertion systems developed for β-lactamase [21], or split and circular permutation GFP folding assays which only require a short 15 amino acid tag[22–24]. In conjunction with fluorescence-activated cell sorting and DNA sequencing, multiplexed expression assays could be performed with a broad range of organisms and genes. However, it is likely that each technique has its own biases and caveats. Ideally, different reporter systems would be run in parallel and the discrepancies dealt with statistically in the design ranking process.

This methodology joins a suite of technologies that employ next generation sequencing as an analytical tool to map functional information to genetic ‘parts’. Existing strategies include methods for mapping out the secondary structure of an RNA [25], characterizing strength of promoters and ribosome binding sites [26], and characterizing degron mutants [27]. Our study extends on these studies and demonstrates that next generation sequencing can be employed to measure soluble protein expression. As expression is a necessary prerequisite for molecular function, this methodology is a practical first step in any effort for mining a library of genes for enzyme function, protein:protein interactions, protein:DNA specificity, and the like. The acquisition and sharing of large volumes of expression data will be useful for the synthetic biology community, and may help enable computational prediction of protein expression.

## Supporting Information

S1 Fig Soluble and Insoluble Protein Fraction for Individual Genes. Genes were cloned as C-terminal FLAG tag fusions without the ssTorA or β-lactamase fusion and expressed with an inducible arabinose promoter. Soluble protein was recovered, then the insoluble protein fraction was equalized to the same volume with lysis buffer, then equal volumes of each fraction were loaded on the gel. Western blotting was performed with a monoclonal Anti-FLAG M2-HRP antibody. One representative blot is shown of three.
(TIFF)

S2 Fig Survival of Individual Genes in Expression Correction Assay. Individual gene chimeras with ssTorA and β-lactamase were growth to log phase diluted overnight saturated cultures 1:100 and growing 5h at 30°C. Serial dilutions of cultures were made with 2YT media and 5μL was spotted on solid agar media with chloramphenicol and 0 or 100 μg/mL ampicillin and incubated for 16h at 30°C. Fold killing is calculated by counting the number of cells at 100 μg/mL and dividing by the count at 0 μg/mL. One representative experiment is shown of three.
(TIFF)

S1 Dataset GFP Chip Oligo Sequences. Contains the oligo sequences for the GFP chip.
(TXT)

S2 Dataset GFP Chip Gene Sequences. Contains the gene sequences for the GFP chip.
(TXT)

S3 Dataset BRENDA Chip Oligo Sequences. Contains the oligo sequences for the BRENDA characterization chip.
(TXT)

S4 Dataset BRENDA Chip Gene Sequences. Contains the gene sequences for the BRENDA characterization chip.
(TXT)

S1 Table Gene Counts. Contains the median counts per gene from NGS of post-selection plasmids.
(XLSX)
