# Correction: A Method for Multiplex Gene Synthesis Employing Error Correction Based on Expression

**DOI:** 10.1371/journal.pone.0126078

**Published:** 2015-05-06

**Authors:** Timothy H.-C. Hsiau, David Sukovich, Phillip Elms, Robin N. Prince, Tobias Strittmatter, Paul Ruan, Bo Curry, Paige Anderson, Jeff Sampson, J Christopher Anderson

The fifth author’s name is spelled incorrectly. The correct name is Tobias Strittmatter. The correct citation is:

Hsiau TH-C, Sukovich D, Elms P, Prince RN, Strittmatter T, Ruan P, et al. (2015) A Method for Multiplex Gene Synthesis Employing Error Correction Based on Expression. PLoS ONE 10(3): e0119927. doi:10.1371/journal.pone.0119927
