# Supplementary figures and images for: A Method for Multiplex Gene Synthesis Employing Error Correction Based on Expression
Source: PLoS One. 2015 Mar 19;10(3):e0119927. doi: 10.1371/journal.pone.0119927 (PMC4366238; doi:10.1371/journal.pone.0119927)

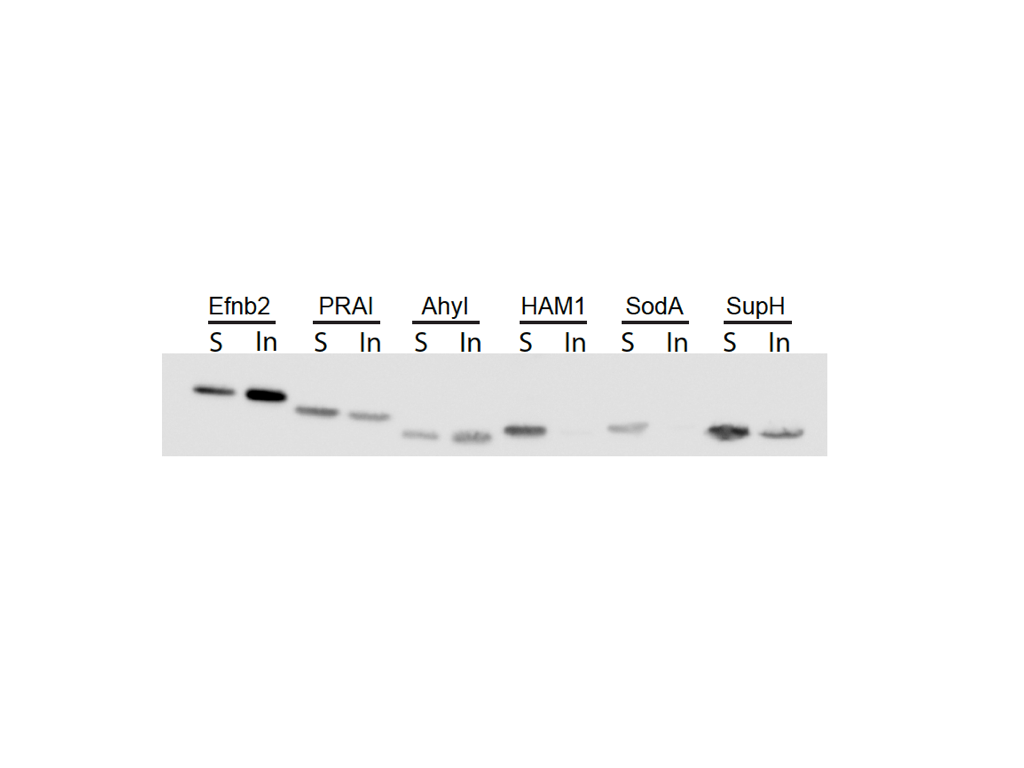

Supplement: S1 Fig — Genes were cloned as C-terminal FLAG tag fusions without the ssTorA or β-lactamase fusion and expressed with an inducible arabinose promoter. Soluble protein was recovered, then the insoluble protein fraction was equalized to the same volume with lysis buffer, then equal volumes of each fraction were loaded on the gel. Western blotting was performed with a monoclonal Anti-FLAG M2-HRP antibody. One representative blot is shown of three. (TIFF) [file pone.0119927.s001.tiff]

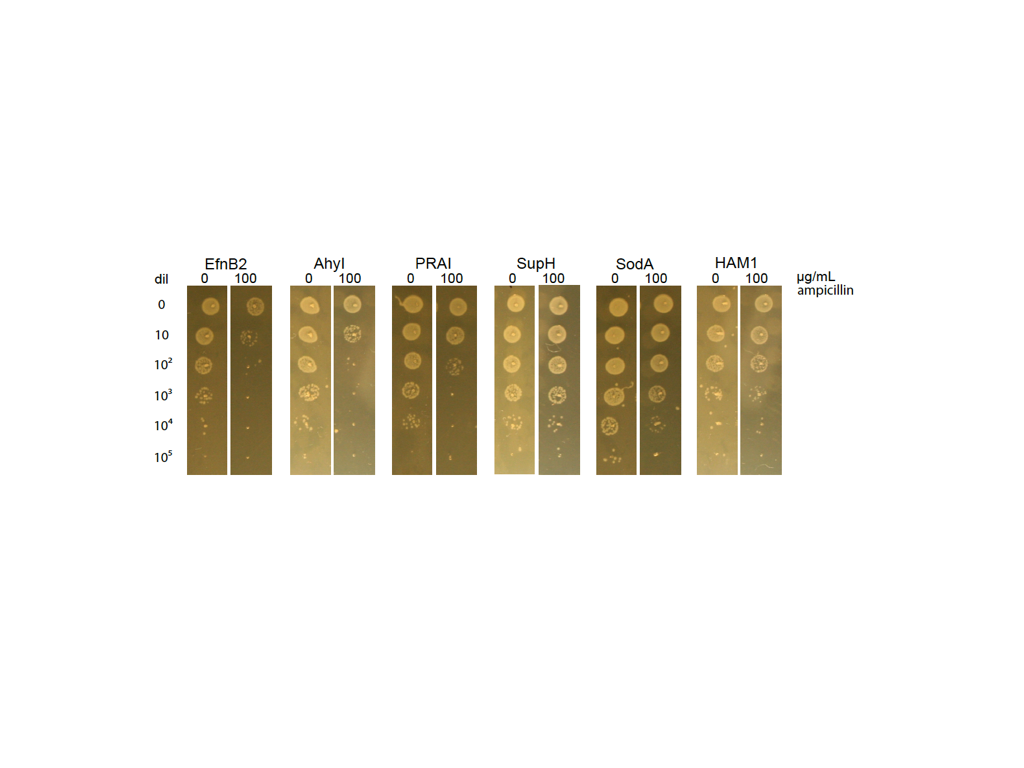

Supplement: S2 Fig — Individual gene chimeras with ssTorA and β-lactamase were growth to log phase diluted overnight saturated cultures 1:100 and growing 5h at 30°C. Serial dilutions of cultures were made with 2YT media and 5μL was spotted on solid agar media with chloramphenicol and 0 or 100 μg/mL ampicillin and incubated for 16h at 30°C. Fold killing is calculated by counting the number of cells at 100 μg/mL and dividing by the count at 0 μg/mL. One representative experiment is shown of three. (TIFF) [file pone.0119927.s002.tiff]
